# Risk factors for posttransplant lymphoproliferative disorder (PTLD) in pediatric heart recipients were associated with impairment of the immune system – a multicenter study from Sweden

**DOI:** 10.1016/j.jhlto.2026.100673

**Published:** 2026-08-27

**Authors:** Britt-Marie Ekman-Joelsson, Michal Odermarsky, Karin Tran-Lundmark, Maria Sjöborg-Alpman, Katarina Lannering, Bengt Andersson, Clas Malmeström, Håkan Wåhlander, Karin Mellgren, Olov Ekwall

**Affiliations:** a Pediatric Heart Centre, Sahlgrenska University Hospital, Region Västra Götaland, Gothenburg, Sweden; b Department of Pediatrics, Institute of Clinical Sciences, The Sahlgrenska Academy, University of Gothenburg, Gothenburg, Sweden; c Pediatric Heart Center, Skåne University Hospital; Pediatric Cardiology, Department of Clinical Sciences Lund, Lund University, Lund, Sweden; d Pediatric Heart Center, Skåne University Hospital and Department of Experimental Medical Science and Wallenberg Center for Molecular Medicine, Lund University, Lund, Sweden; e Pediatric Heart Center, Stockholm-Uppsala, Karolinska University Hospital, and Department of Children's and Women's Health, Karolinska Institutet, Stockholm, Sweden; f Department of Clinical Immunology and Transfusion Medicine, Sahlgrenska University Hospital, Gothenburg, Sweden; g Department of Rheumatology and Inflammation Research, Department of Pediatrics, Institute of Medicine, The Sahlgrenska Academy, University of Gothenburg, Gothenburg, Sweden; h Department of Pediatric Oncology, Sahlgrenska University Hospital, Region Västra Götaland, Gothenburg, Sweden

**Keywords:** pediatric heart transplantation, post-transplant lymphoproliferative disorder, thymectomy, congenital heart disease, immune system

## Abstract

**Background:**

Posttransplant lymphoproliferative disorder (PTLD) is a potentially lethal complication after pediatric heart transplantation (PHT). We have previously reported an association between PTLD and (1) sternotomy during infancy, (2) mismatch concerning Epstein–Barr virus infection, (3) hypoplastic left ventricle and (4) surgically palliated congenital heart disease (CHD) as transplant indication versus cardiomyopathy (CMP). To explore whether there were significant differences in immunological profiles between the identified risk groups for PTLD and the rest of pediatric heart recipients.

**Methods:**

Data was partially collected as registry data from the previous study concerning PHT, PKT and HC 7. We then added clinical data and prospectively collected blood samples from additional PHT subjects from the 3 Swedish tertiary centers performing long-term follow-up after PHT. Blood samples were collected after transplantation (mean time 4.6 years, range 0.2–14.7 years) and were analyzed for immunophenotyping of T- and B-lymphocyte subpopulations and monocytes by flow cytometry, and T-cell receptor excision circles (TREC).

**Results:**

A total of 76 PHT subjects were included. Thirty-eight had undergone thymus excision during infancy and 22 had hypoplastic left ventricle. The transplant indication was CHD in 36 and CMP in 40 children. The immune profiles of the identified risk groups for PTLD were characterized by low numbers of TREC, naïve CD4+, naïve CD8+, naïve recent thymic emigrants (RTE) and among patients with CHD low numbers of T-helper cells (Th). In addition, the relative proportions of naïve cells were low.

**Conclusions:**

The identified PTLD risk groups demonstrated significant impairment in the immune system, a pattern consistent with immune dysfunction and features of immunological aging, both of which are conditions known to increase the risk of developing PTLD.

## Background

Posttransplant lymphoproliferative disorder (PTLD) remains a serious complication and a therapeutic challenge after pediatric heart transplantation.^1^ In more than 90% of humans, EBV is incorporated into B-lymphocytes and controlled by T-cells. In immunocompromised individuals, where T-cell control is impaired, the virus can induce uncontrolled B-cell proliferation resulting in PTLD.^2^ Immunosuppression is generally accepted as the primary cause of EBV-induced PTLD. The use of induction therapy does, however, not correlate to an increased rate of PTLD.^3^ The understanding that different immunosuppressive regimens account for different rates or PTLD was challenged by a recent study, in which no associations were found between PTLD and transplant induction therapy or protocols including tacrolimus.^4^ The Pediatric Heart Center in Gothenburg faced an increased rate of PTLD and a retrospective analysis unveiled the following risk factors for PTLD; (1) sternotomy during infancy; (2) mismatch concerning Epstein–Barr virus infection, (3) hypoplastic left ventricle and (4) surgically palliated CHD.^5^ As early sternotomy was the strongest risk factor, we suspected a more complex etiology of PTLD than just immune suppression and EBV-mismatch.^6^ We conducted a cross-sectional study of the immune system among pediatric heart transplanted (PHT) subjects in Gothenburg. To further understand the impact of immunosuppression, a control group of pediatric kidney transplanted (PKT) subjects was added, in addition to healthy controls (HC). The main finding of this study was that the immune system was activated after PKT, while it was downregulated after PHT.^7^ To be able to explore differences regarding immune function between the sub-groups of PHT subjects, the study had to be expanded to a national study. The aim of the present study was to explore whether there were significant differences in immunological profiles between the identified risk groups for PTLD and the rest of pediatric heart recipients.

## Material and methods

Data was partially collected as registry data from the previous study concerning PHT, PKT and HC.^7^ We then added clinical data and prospectively collected blood samples from additional PHT subjects from the 3 Swedish tertiary centers performing long-term follow-up after PHT. Demographic data, indications for transplantation, CHD type, and timing of thymus excision were collected from medical records. Data concerning EBV-serology was collected from YASWA (Scandia transplants database) and for infants from the virology laboratory at Sahlgrenska University Hospital. The EBV-viral load was not focused as the correlation of the levels in specific subjects at a given time and the risk of PTLD is not clearly understood.^8^, ^9^

The study was approved by the Central Ethical Review Board at the University of Gothenburg (Dnr. 243-17). Written informed consent was obtained from the participants and their caretakers. Donor organs used for heart transplantation were procured with autonomous consent free from coercion, consistent with the Declaration of Istanbul and the International Society for Heart and Lung Transplantation (ISHLT) Statement on Transplant Ethics.

All analyses were performed at the Department of Clinical Immunology and Transfusion Medicine, Sahlgrenska University Hospital, Gothenburg, according to standard operating procedures; see Supplementary material. Fresh whole blood was used to determine absolute numbers and proportions of T and B lymphocytes, natural killer cells, myeloid cells, and subpopulations thereof, as proposed by the Human Immunophenotyping Consortium with minor modifications.^10^ For T-cell receptor excision circles (TREC), DNA was extracted from fresh whole blood using the QIAamp Blood Mini Kit (Qiagen, Venlo, the Netherlands), followed by a triplex real-time quantitative PCR for TREC and the endogenous reference gene glyceraldehyde-3-phosphate dehydrogenase.^11^ Table S1 shows the characteristics of the different immune cell types.

## Statistical methods

Demographic and immunological variables were compared using the Mann–Whitney *U* test and Fisher’s exact test, as appropriate. *P* values were adjusted for multiple testing using the Benjamini–Hochberg procedure, a statistical method used to control the False Discovery Rate (FDR) when performing multiple hypothesis tests simultaneously. The threshold for significance was an adjusted *p*-value (*p*-adj) of <0.05 based on a False Discovery Rate (Q) of 5%.^12^ All analyses were performed in R version 4.0.2.

## Results

A total of 76 subjects, heart transplanted in Gothenburg and Lund (6.6 weeks−17.7 years of age, median 5.6 years), were included in the study, recruited from 3 tertiary centers in Sweden: Gothenburg (*n* = 49), Lund (*n* = 21) and Stockholm (*n* = 6). There was patient overlap between the studies from Gothenburg, for details see Table 1. Blood samples were obtained at 1 month–14.5 years (median 6.2 year) after heart transplantation and in connection with regular follow-up, including evaluation of rejection and exclusion of infection. One subject was excluded, due to a rare genetic disorder, a mutation in the RTTN-gene, including microcephaly and multiple endocrine disorders. No genetic testing was performed. Results from blood analysis from PHT subjects were compared with results from blood analysis in 34 age- and sex-matched PKT subjects and 33 HC described in a previous study.^7^

**Table 1:** Description of Patient Overlaps Between the Three Studies

	Study design	Total cohort of pediatric heart recipients	Overlap from previous study	Subjects with PTLD
Study published 2017	Retrospective	71	None	10
Study published 2023	Prospective	36	25	5
Present study	Prospective	76	36	6

PTLD, post-transplant lymphoproliferative disease.

Indications for transplantation were CHD (*n* = 36) and CMP (*n* = 40). See Table 2 for demographics. It was not possible to obtain consistent data regarding the extent of thymus excision. The term thymectomy is only used when it is clearly described as thymectomy. Thymus excision had been performed in infancy, (1 day–1 year, median 2.4 months of age) in 38 subjects, and in 37 subjects after 1 year of age (1.4–17.3 years of age, median 10.7 year). Of the 38 who had undergone thymus excision before 1 year of age, transplant indication was CMP in 7 and CHD in 31. See Table 3a for type of CHD, in relation to univentricular circulation, see Table 3b for type of CHD in relation to time of thymus excision. In the subgroup of 22 subjects with CHD involving a hypoplastic left ventricle, all had undergone thymus excision, performed between 1 and 51 days of age (median 8 days). See Table 4 for subtypes of cardiomyopathy and timing of thymus excision. The group of PHT subjects, divided into transplant indication CHD and CMP and PKT subjects were similar regarding age, sex and time between transplantation and blood sample, allowing for comparison of the data from blood samples.

**Table 2:** Baseline Demographics of the Subjects, Time Between Transplantation and Blood Sample and Age at Transplantation

	CHD (*n* = 36)	CMP (*n* = 40)	KTX (*n* = 34)	Healthy controls (*n* = 33)
Age, years, (median range)	13.7 (3.6–22.2)	12.9 (1.4–21.6)	12.8 (3.3–16.7)	13.0 (2.5–24)
Gender, female/male, *n*	15/21	20/20	11/23	11/22
Time between transplantation and blood sample, years (median, range)	5.3 (0.2–14.7)	4.0 (0.1–13.8)	6.1 (0.2–15.0)	
Age at transplantation, years (median, range)	8.9 (0.5 – 17.7)	9.4 (0.07 – 17.0)	6.0 (1.0 – 16.0)	

CHD, congenital heart disease; CMP, cardiomyopathy; KTX, kidney transplantation.

**Table 3a:** Specific Heart Defects

Heart defect	Number	Hypoplastic left heart	Fontan circulation	PLE/plastic bronchitis	Univentricular circulation
HLHS	14	14	7	3	14
Unbalanced AVSD	5	5	3	2	5
DORV/DOLV	4	3	1	0	4
TA	3	0	2	0	3
DILV/TGA/CoA	1	0	0	0	1
PAIVS	1	0	0	0	1
AS	2	0	0	0	0
LTGA	2	0	0	0	0
AAOLCA	1	0	0	0	0
HRV	1	0	0	0	0
CoA/VSD	1	0	0	0	0
AVSD/PS	1	0	0	0	0

AAOLCA, Anomalous Aortic Origin of the Left Coronary Artery; AS, aortic stenosis; AVSD, atrioventricular septal defect; CoA, coarctation; DILV, double inlet left ventricle; DOLV, double outlet left ventricle; DORV, double outlet right ventricle; HLHS, hypoplastic left heart syndrome; HRV, hypoplastic right ventricle; LTGA, Levo-transposition of the great arteries; PAIVS, pulmonary atresia and intact ventricular septum; PLE, protein losing enteropathy; PS, pulmonary stenosis; TA, tricuspid atresia; TGA, transposition of the great arteries; VSD, ventricular septal defect.

**Table 3b:** Specific Heart Defects in Relation to Age of Thymus Excision

Heart defect	Number	Age at thymus excision Range (median) days
HLHS	14	1–15 (median 8)
Unbalanced AVSD	5	7–70 (median 50)
DORV/DOLV	4	5–44 (median 18)
TA	3	43; 384 (m.d.1)
DILV/TGA/CoA	1	7
PAIVS	1	23
AS	2	7; 12
LTGA	2	3,696 (m.d. 1)
AAOLCA	1	6,030
HRV	1	1,318
CoA/VSD	1	8
AVSD/PS	1	520

AAOLCA, Anomalous Aortic Origin of the Left Coronary Artery; AS, aortic stenosis; AVSD, atrioventricular septal defect; CoA, coarctation; DILV, double inlet left ventricle; DOLV, double outlet left ventricle; DORV, double outlet right ventricle; HLHS, hypoplastic left heart syndrome; HRV, hypoplastic right ventricle; LTGA, Levo-transposition of the great arteries; m.d., missing data; PAIVS, pulmonary atresia and intact ventricular septum; PS, pulmonary stenosis; TA, tricuspid atresia; TGA, transposition of the great arteries; VSD, ventricular septal defect.

**Table 4:** Type of Cardiomyopathy in Relation to Time of Thymus Excision

Type of cardiomyopathy	Number	Age at thymus excision Range (median) days
Dilated cardiomyopathy	28	25–6,266 (median 135)
Restrictive cardiomyopathy	5	856–4,202 (median 3182)
Hypertrophic cardiomyopathy	7	282 – 5,264 (median 2767)

### EBV-serology and mismatch

Nine PHT subjects were below 1 year of age at transplantation (25 days–9 months, median 6 months), 7 had CMP as transplant indication, 2 had CHD. Five had IgG antibodies against EBV but as there were no symptoms of EBV, the positive IgG antibodies were interpreted as likely maternally derived antibodies. Four of the 9 donors were IgG positive. Among the other 67 PHT subjects 26 had mismatch (missing data in 2 subjects), where the donor was positive and the recipient negative for EBV. See Table 5.

**Table 5:** EBV-Serology

HTX, age (year)	IgG donor, positive	IgG donor negative	IgG recipient positive	IgG recipient negative	Possible Mismatch
<1	4	5	5	4	4
>1	46 (m.d. 9)	12 (m.d. 9)	33 (m.d.1)	33 (m.d.1)	26

HTX, Heart transplantation; IgG, Immunoglobulin G; m-d-, missing data; mismatch, igG positive donor and IgG negative recipient.

### Immunosuppression

Table S2 summarizes the transplantation-related medications. There were slight differences concerning induction therapy between centers, anti-thymocyte globulin was the standard treatment in Lund during the whole time and in Gothenburg until 2015, while basiliximab was standard in Gothenburg from 2015 onward. Surgery was not performed in Stockholm. The protocols for follow-up were similar for all subgroups of PHT but with minor differences between centers, for example were target levels for tacrolimus slightly higher in Lund compared with Gothenburg. For maintenance therapy tacrolimus was the drug of choice, in combination with mycophenolate mofetil (MMF). The protocol for PKT differed slightly, with less immunosuppression during induction, but similar at follow-up, though steroid treatment was more frequent after PKT. See Table S2 (Supplementary material).

### TREC

**Figure 1:**
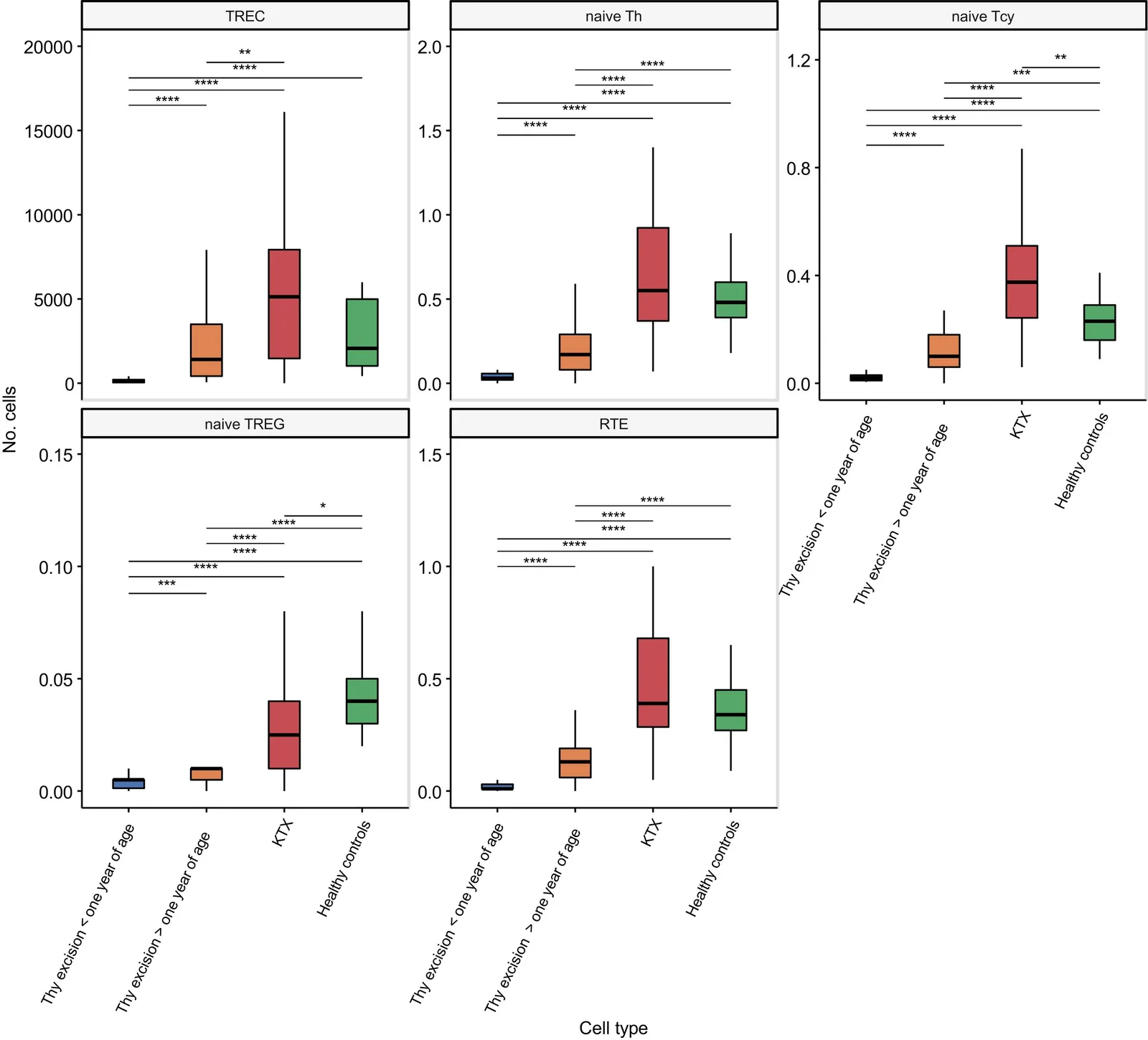

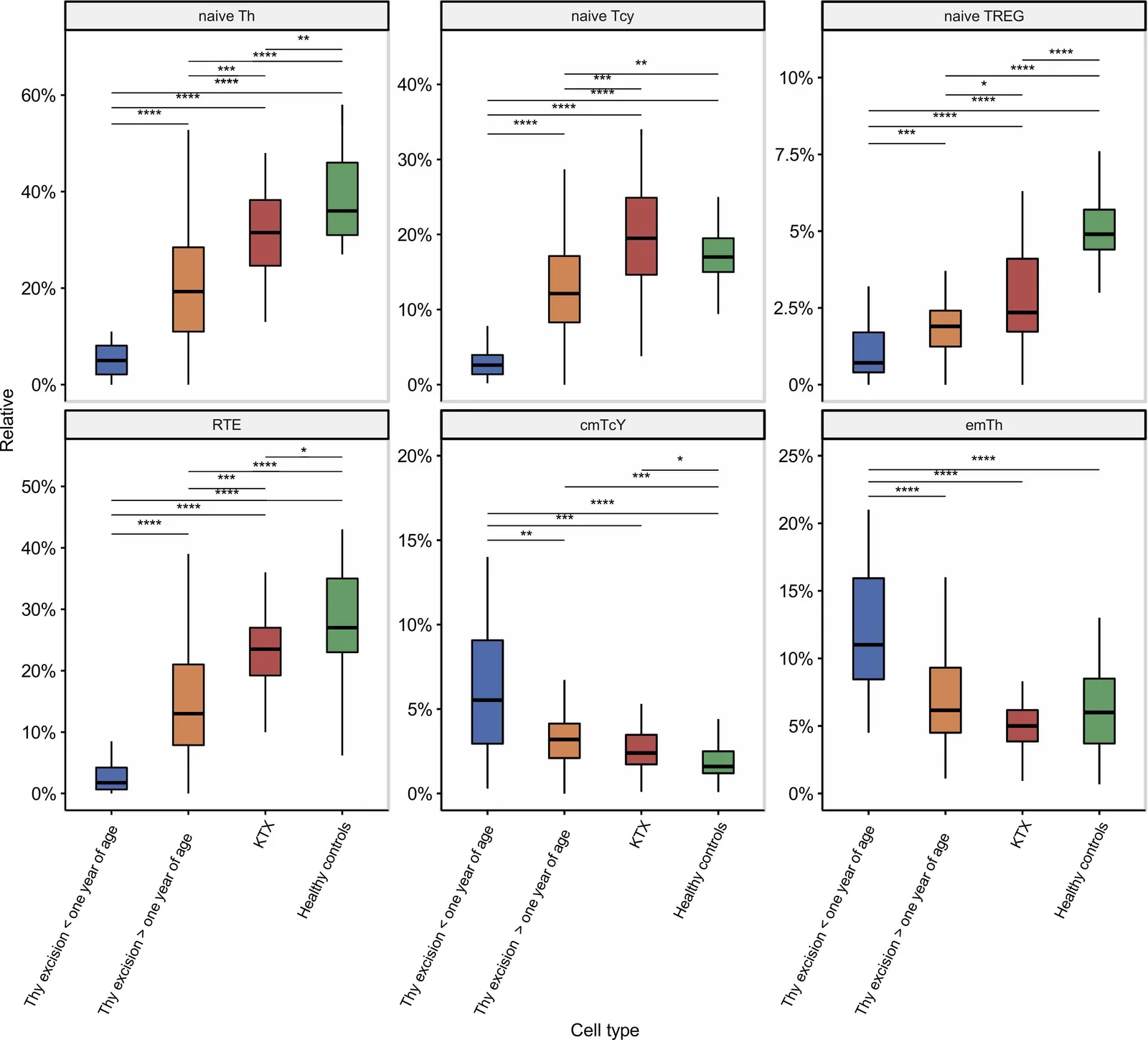
(a) Absolute numbers of T-cells, focusing on Thymus excision. Thy = thymus. Thymus excision < 1 year of age (*n* = 38) performed 1 day–1 year of age (median 2.4 months), Thymus excision > 1 year of age (*n* = 37) performed at 1.4–17.3 years of age (median 10.7 year), KTX = Kidney transplanted (*n* = 34), Healthy controls (*n* = 33). TREC = T-cell Receptor Excision Circle, Th = T helper cells, Tcy = Cytotoxic T-cells, TREG = T-Regulatory cells, RTE = Recent Thymic Emigrants. For type of heart defects and type of cardiomyopathy in relation to thymus excision see Tables 3a, 3b and 4. (b) Relative numbers of T-cells, focusing on Thymus excision. Thy = thymus. Thymus excision < 1 year of age (*n* = 38) performed 1 day–1 year of age (median 2.4 months), thymus excision > 1 year of age (*n* = 37) performed at 1.4–17.3 years of age, (median 10.7 year), KTX = Kidney transplanted (*n* = 34), Healthy controls (*n* = 33). Th = T helper cells, Tcy = Cytotoxic T-cells, TREG = T-Regulatory cells, RTE = Recent Thymic Emigrants, cm Tcy = activated central memory cytotoxic T-cells, em Th = effector memory T-helper cells. For type of heart defects and type of cardiomyopathy in relation to thymus excision see Tables 3a, 3b and 4.

The TREC numbers were significantly lower in all the previously identified risk groups for PTLD, sternotomy below 1 year of age, hypoplastic left ventricle and CHD as transplant indication, compared to the whole PHT group and healthy controls. The PKT group had higher levels of T-cell receptor excision circles than the healthy control group reflecting an activated immune system. See Figure 1.

### T-cells

We present both absolute and relative numbers for all lymphocyte subsets respectively, since the functional impact of absolute versus relative lymphocyte subsets is not clear. We focus the analysis on absolute numbers in the major lymphocyte populations and relative numbers in the smaller subpopulations.

PHT subjects who had undergone thymus excision before 1 year of age compared with thymus excision after 1 year of age had significantly lower absolute numbers of naïve CD8+ (cytotoxic T-cells), naïve CD4+ (T-helper cells), naïve regulatory T-cells (Treg), recent thymic emigrants (RTE) and CD3+ T-cells, higher numbers of activated effector memory CD3+, and activated effector memory CD8+. Relative numbers of CD3+, naïve CD4+, naïve CD8+, naïve Treg and recent thymic emigrants (RTE) were also lower after early thymus excision, while memory T-cells were higher in heart transplanted children with early thymus excision. PKT had higher numbers of naïve CD8+ (cytotoxic T-cells), naïve CD4+ (T-helper cells). See Figures 1a and 1b.

**Figure 2:**
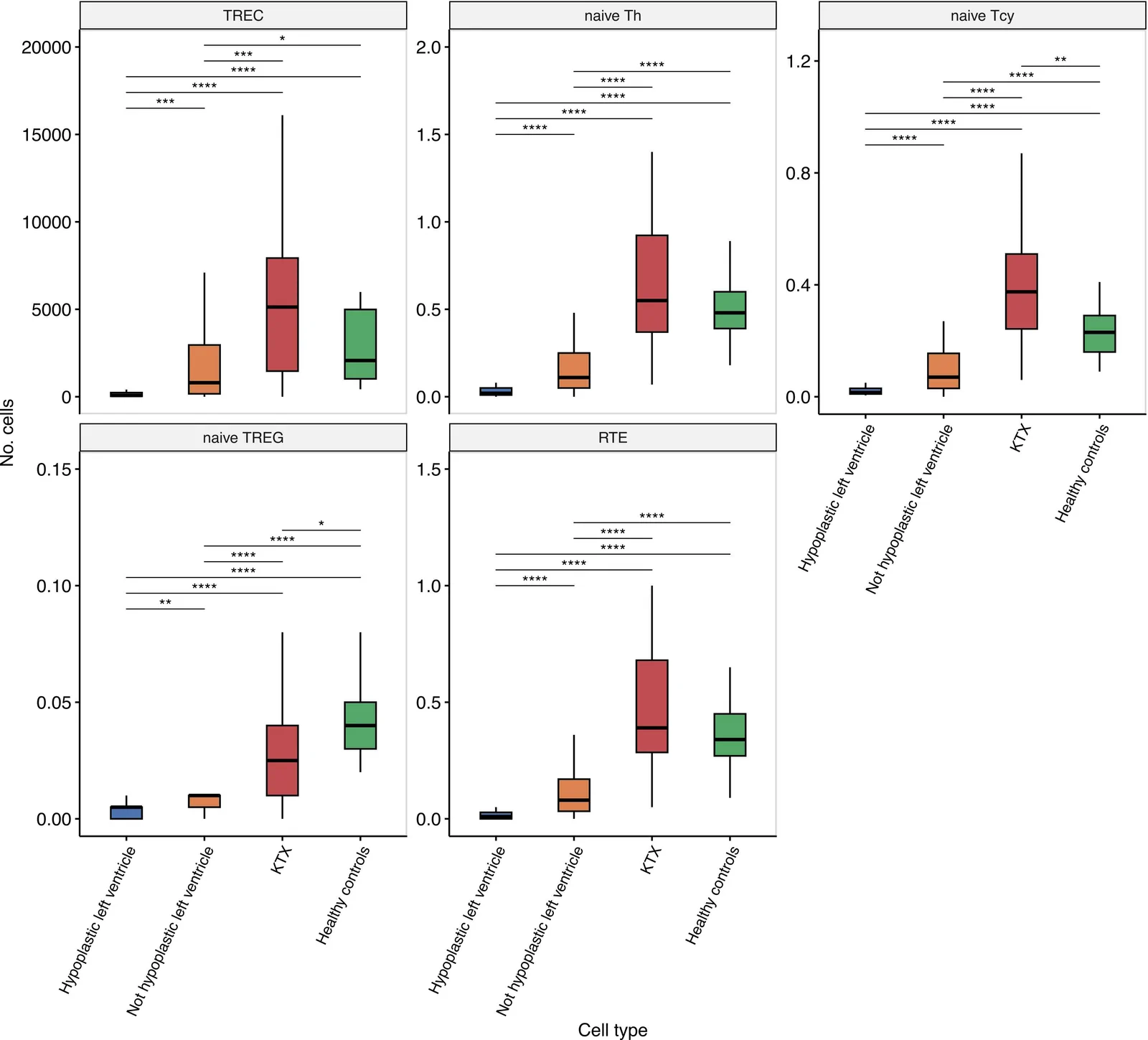

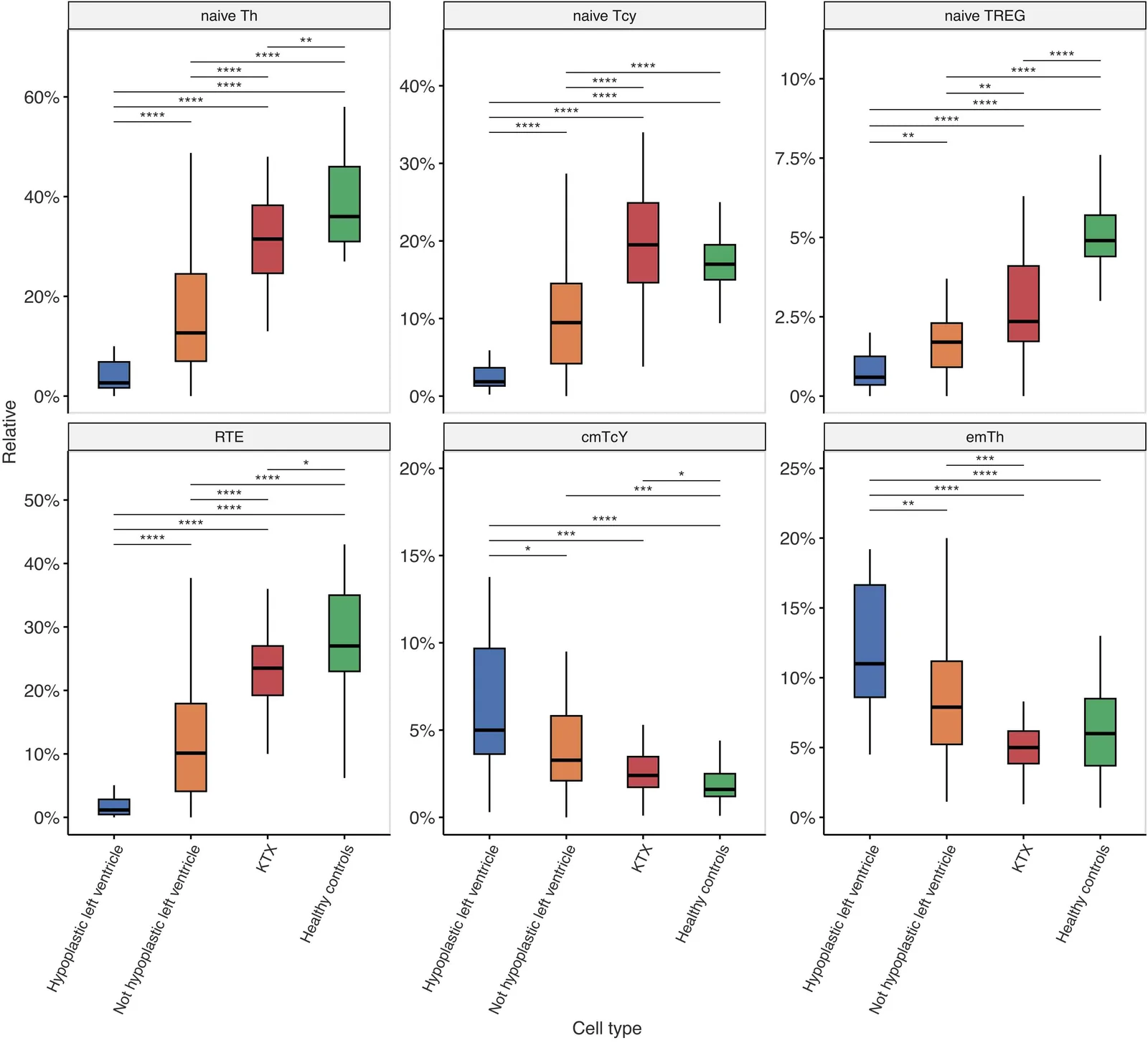
(a) Absolute numbers of T-cells focusing on hypoplastic left ventricle (*n* = 22) in whom thymus excision were performed at 1–51 days of age (median 8 days), not hypoplastic left ventricle (*n* = 54), in whom thymus excision were performed at 7–6,266 days of age (median 3,206 days), KTX = Kidney transplanted (*n* = 34), Healthy controls (*n* = 33). TREC = T-cell Receptor Excision Circle, Th = T helper cells, Tcy = Cytotoxic T-cells, TREG = T-Regulatory cells, RTE = Recent Thymic Emigrants. For type of heart defects and type of cardiomyopathy and in relation to thymus excision see Tables 3a, 3b and 4. (b) Relative numbers of T-cells focusing on hypoplastic left ventricle (*n* = 22) in whom thymus excision were performed at 1–51 days of age (median 8 days), not hypoplastic left ventricle (*n* = 54), in whom thymus excision were performed at 7–6,266 days of age (median 3,206 days), KTX = Kidney transplanted (*n* = 34), Healthy controls (*n* = 33). Th = T helper cells, Tcy = Cytotoxic T-cells, TREG = T-Regulatory cells, RTE = Recent Thymic Emigrants, cm Tcy = activated central memory cytotoxic T-cells, em Th = effector memory T-helper cells. For type of heart defects and type of cardiomyopathy and in relation to thymus excision see Tables 3b and 4.

PHT subjects with hypoplastic left ventricle had significantly lower amounts of CD3+, naïve CD4+, naïve CD8+, naïve Treg and RTE, than all the other groups. Furthermore, their relative numbers of naïve CD4+, naïve Treg and RTE were lower, but higher for naïve CD8+, cmCD8+ and effector memory T-cells. See Figures 2a and 2b.

**Figure 3:**
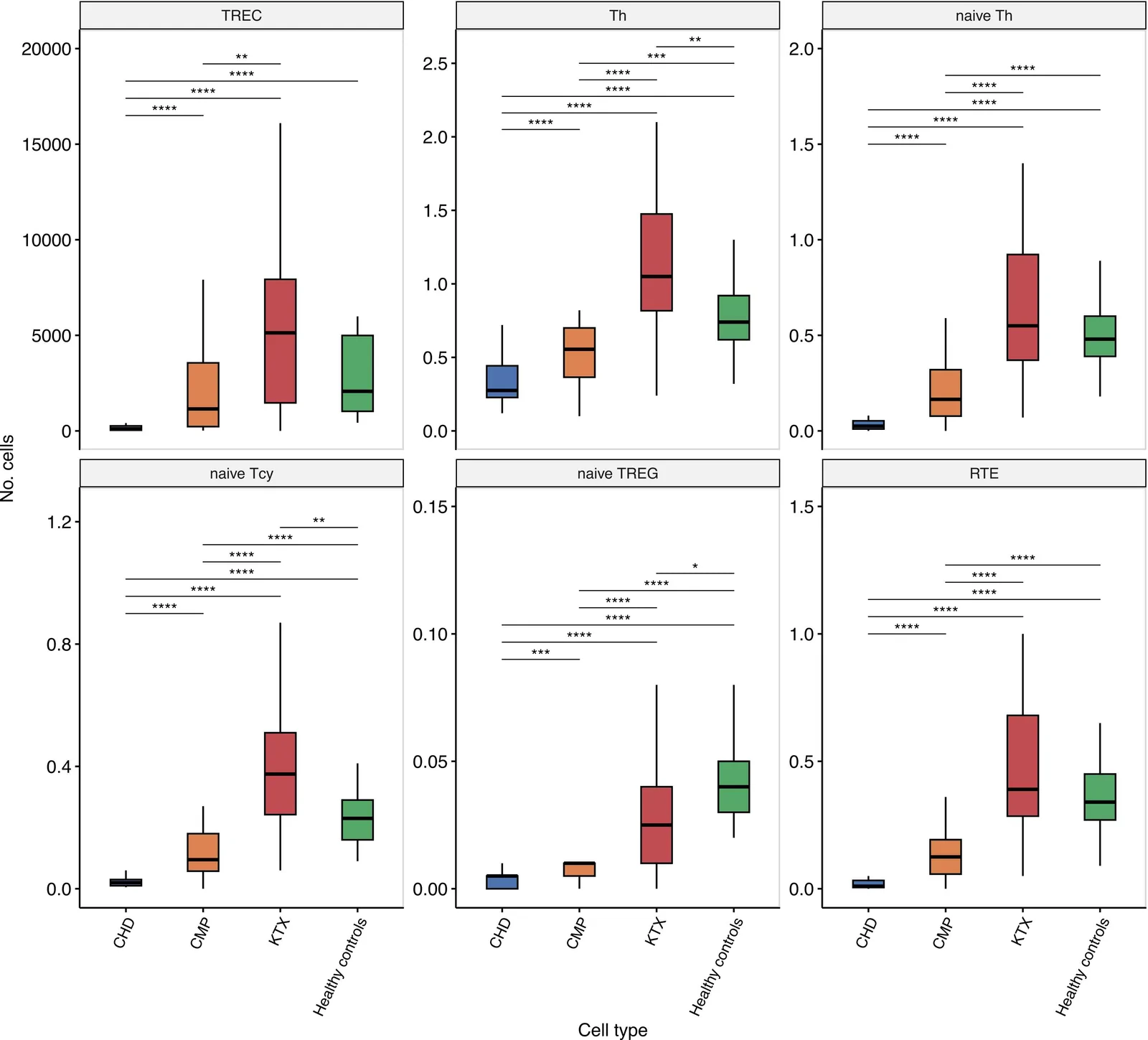

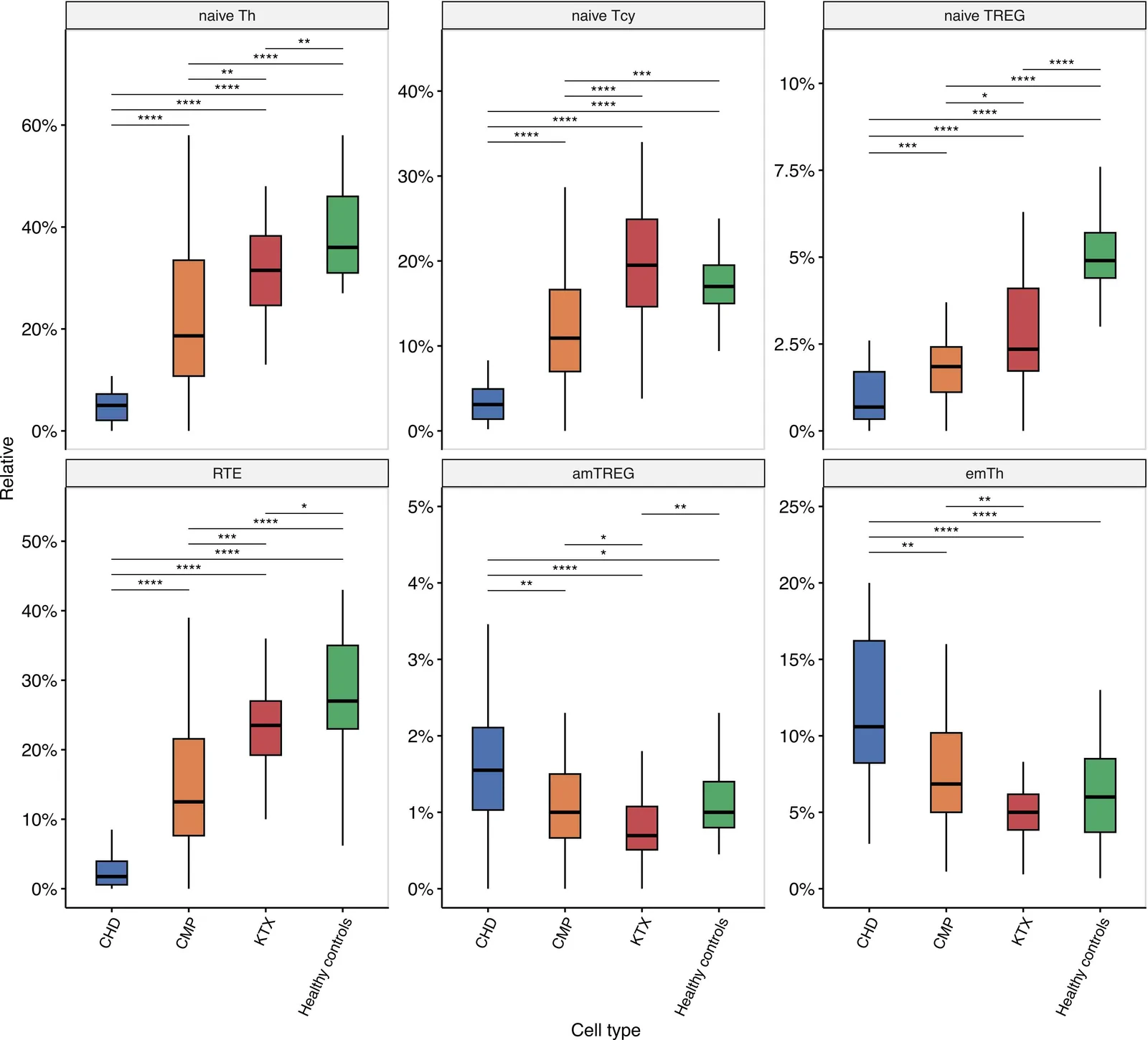
(a) Absolute numbers of T-cells focusing on congenital heart disease (*n* = 36) in whom thymus excision was performed at 1–6,036 days of age (median 11 days), versus cardiomyopathy (*n* = 40) in whom thymus excision was performed at 25–6,266 days of age (median 3,465 days) as transplant indication, CHD = congenital heart disease, CMP = cardiomyopathy, KTX = Kidney transplanted (*n* = 34), Healthy controls (*n* = 33). TREC = T-cell Receptor Excision Circle, Th = T helper cells, Tcy = Cytotoxic T-cells, TREG = T-Regulatory cells, RTE = Recent Thymic Emigrants. For type of heart defects and type of cardiomyopathy and in relation to thymus excision see Tables 3a, 3b and 4. (b) Relative numbers of T-cells focusing on congenital heart disease (*n* = 36) in whom thymus excision was performed at 1–6,036 days of age (median 11 days), versus cardiomyopathy (*n* = 40) in whom thymus excision was performed at 25–6,266 days of age (median 3,465 days), as transplant indication, KTX = Kidney transplanted (*n* = 34), Healthy controls (*n* = 33). Th = T helper cells, Tcy = Cytotoxic T-cells, TREG = T-Regulatory cells, RTE = Recent Thymic Emigrants, cm Tcy = activated central memory cytotoxic T-cells, em Th = effector memory T-helper cells. For type of heart defects and type of cardiomyopathy and in relation to thymus excision see Tables 3a, 3b and 4.

When comparing groups based on transplant indication CHD in comparison with CMP there were significantly lower amounts of TREC, T-cells, CD3+, naïve CD4+, naïve CD8+, naïve Treg and RTE in the CHD group, while the relative numbers also were low concerning naïve CD3, naïve Treg and RTE, higher for naïve CD8+, activated memory Treg and memory CD3. See Figures 3a and 3b. Comparing the group with CHD as transplant indication with healthy controls the same pattern was seen, very low amounts of TREC, naïve CD4+, naïve CD8+, naïve Treg, memory Treg, activated memory Treg, CD3+, RTE, double negative cells, lymphocytes, and central memory CD8+. Circulating T follicular helper cells were however present in higher amounts in PHT subjects, when CHD was the transplant indication compared to healthy controls. The relative numbers of naïve CD4+, naïve CD8+, naïve Treg, RTE, CD3 and lymphocytes were lower while different memory T-cells had higher relative numbers.

### B-cells

The total number of B-cells was lower among PHT subjects compared to PKT subjects. There were no differences in total numbers of B-cells between subgroups of heart transplanted children. Among subgroups of B-cells the only difference was higher numbers of plasmablasts after early thymus excision.

PHT subjects with CHD had significantly lower absolute and relative numbers of IgM memory B-cells, class-switched memory B-cells and lower absolute numbers of transitional B-cells, in comparison with HC, as well as with PKT subjects.

### PTLD

In our cohort of 76 patients, 6 patients developed PTLD after 0*.*4–15 (median 0.6) years post-transplant. All 6 had CHD as transplant indication and 5 had hypoplastic left ventricle. Sternotomy had been performed at 5–44 days of age (median 7 days). In 3 cases there was a complete thymectomy, in 2 thymus excision was performed and in one data was missing. The subjects were between 7 and 17 years of age (median 15) at the time of PTLD diagnosis and time after transplantation was 0.5–16.7 years (median 11.1 years). One subject was transplanted below 1 year of age, no mismatch concerning EBV as the donor was IgG negative, the patient was however thymectomized at 5 days of age, had hypoplastic left heart syndrome as transplant indication. PTLD occurred at 15 years of age. In 5 cases the recipient was EBV-negative and mismatch occurred in 3 cases. The immune profile was similar to the CHD group. There were no occurrences of PTLD among the PKT group.

## Discussion

In heart-transplanted children with characteristics previously identified as risk factors for PTLD, congenital heart disease as transplant indication, hypoplastic left heart, and thymus excision before 1 year of age, we found evidence of a significantly impaired immune system, especially in the T-cell compartment, compared with kidney-transplanted children and healthy controls. An impaired T-cell compartment may explain the increased risk of PTLD in this group. The pattern of immune dysfunction is consistent with thymic dysfunction and immunological aging secondary to early thymus excision. A prerequisite for EBV-induced lymphoma (PTLD) is a dysfunction of the immune system.^13^ EBV mismatch between donor and recipient is a well-known strong risk factor for PTLD, highlighting the importance of the host immune response.^6^ In a large register study, Pediatric Heart Transplant Study, younger age at heart transplantation was an even stronger risk factor for late PTLD than EBV-mismatch.^14^ EBV-mismatch could have been underestimated as it is based on serology and in infancy antibodies can emanate from the mother’s blood. In our cohort 9 subjects had been transplanted below 1 year of age, only 5 had antibodies against EBV, a lower number than previously reported.^15^ Younger age at transplantation as a risk factor includes earlier sternotomy and thymus excision which means that the finding is in line with our study.^5^ Historically, the degree of immunosuppression correlates with PTLD risk, but current immunosuppression protocols do not show the same correlation.^4^, ^16^ PTLD is also linked to other immunodeficiency states, including AIDS, primary immunodeficiencies, and advanced biological aging.^17^, ^18^, ^19^ In our cohort, the occurrence of PTLD was likely multifactorial, involving immune responses to Epstein–Barr virus with both T- and B-cell activation. Although our cohort is too small to draw firm conclusions, it is noteworthy that all patients who developed PTLD had undergone thymectomy at a very young age. This observation is consistent with our hypothesis that early loss of thymic function may contribute to PTLD susceptibility, but the finding should be interpreted cautiously given the small sample size and the interrelationship between age, thymectomy and EBV status. Advances in immunology over recent decades have clarified multiple specialized T-cell functions, including a significant role in the defense against virus-induced cancer.^13^ The development of advanced immunotherapies, based on these findings, is a rapidly evolving research area.^20^, ^21^

### Heart transplanted children

PHT subjects have a higher incidence of PTLD compared with PKT subjects (3–28% versus 0.84–3.5% depending on cohort definitions).^1^, ^4^, ^8^, ^9^, ^22^ In a previous study, we found downregulation of the immune system in PHT, irrespective of indication. All heart-transplanted subjects had undergone sternotomy with some form of thymus excision. The immune profile was consistent with impaired thymic function related to thymus excision, a finding not seen in kidney-transplanted controls, despite similar immune suppression.^7^

### Congenital heart disease

Our results demonstrate significantly lower T-cell levels when the transplant indication was CHD compared with CMP. A large study from Newcastle supports CHD as a risk factor for PTLD in heart transplant recipients.^23^ There are contradictory reports, including single-center studies and large register-based studies, where no correlation between PTLD and CHD was demonstrated.^4^, ^13^, ^22^, ^24^ In the Newcastle report, PTLD risk related to earlier age at thymectomy and low CD4+ and CD8+ counts.^23^ Thymic dysfunction in this group of children has been reported in association with heart surgery and early thymus excision, but it may be further accentuated in certain subgroups of heart defects potentially linked to an underlying, yet unrecognized thymic malformation.

In Swedish register-based studies including 89,542 patients with CHD born between 1930 and 2017, the overall cancer risk was 23% higher than in 890,472 matched controls from the Total Population Register, with the highest risk seen in children with CHD and in the most recent birth cohort (1990–2017).^25^ Hematological cancers accounted for 29% of malignancies in CHD compared with 17% in controls, suggesting an increased risk of hematological cancer later in life.^26^

### Hypoplastic left heart

This subgroup was the most homogenous group regarding time for thymus excision and most probably the excision was the most extensive as the structures in the heart are small and the surgical access difficult. The early resection might be the strongest reason for the impaired immune profile in this group. However, our findings might suggest additional vulnerability, beyond early thymus excision, in CHD patients with a hypoplastic left ventricle. Most of these patients had univentricular circulation, few had completed Fontan surgery and only a small number had protein losing enteropathy - conditions that are known to further compromise lymphatic circulation. This raises the suspicion of primary immunological alterations in the immune system, associated with hypoplastic left heart. In addition, there are close embryological relationships between the heart and thymus, with neural crest cells contributing to both organs.^27^, ^28^ Furthermore, reports also indicate immune dysfunction associated with complex congenital heart defects with cyanosis prior to first surgery and thymus atrophy could be linked to high levels of cortisol, an indication of stress.^29^

### Thymus excision

Thymus excision is performed when the thymus blocks access for cardiac surgery and is especially common in infant surgery involving the cardiac outflow tracts. Neonatal thymus excision in humans has been argued to have limited long-term health impact.^30^ It is well known that thymectomy leads to a reduced T-cell compartment, more affected when thymus excision is performed early in life.^31^, ^32^, ^33^ Some studies reported T-cell differences but no clinical effects.^34^ More recent reports describe compromised health after neonatal thymus excision, autoimmune disorders, infections and cancer.^26^, ^35^, ^36^ Increased cancer rates have also been reported after adult thymectomy.^37^ The thymus is crucial for T-cell maturation and selection and the development of cell-mediated immunity, vital for anti-viral and anti-tumor defense.^38^ Aging of the immune system is characterized by a reduction in thymic output, which is seen in a decline in RTEs and naïve cytotoxic T cells.^39^ Naïve T-cells have a lifespan of 5–10 years which explains why the changes after thymus excision sometimes appear late after surgery. Our findings with low RTE and naïve cytotoxic T cells are consistent with immunological changes in human aging. In addition, naïve T-helper cells were low emphasizing the skewed T-cell profile. Immunological aging is associated with diminished vaccine responses, elevated infection risk, cancer incidence, and autoimmune disease.^39^

### Reduced thymic function in the heart-transplanted group versus activated immune function in the kidney-transplanted group

Despite similar immunosuppressive treatment, the PKT group showed an activated T-lymphocyte compartment, while PHT subjects have a lymphocyte profile characterized by reduced or absent thymic function with low numbers of T-cell receptor excision circles and total and naïve T cells, together with immune activation against the allograft. The high absolute numbers of T cells in the PKT group possibly reflect a constant immune stimulation in combination with normal thymic function. The immune profiles of the PHT and PKT groups show signs of chronic immune activation by the allotransplant. Increased immunological activity is a natural response to allotransplantation, and immunosuppressive therapy is administered to control this response in order to accept the transplanted tissue.^7^ Our hypothesis is that the T-cell defect caused by early thymectomy interacts with the defect caused by immunosuppression, thereby significantly increasing the likelihood of developing PTLD after PHT compared to after PKT.

### Limitations

The main limitation is the small sample size of subjects with PTLD, which does not allow for a multivariate analysis. The reutilization of previously published data also results in a variety of biases and may overstate findings unique to the repeatedly used cohort, especially in a study with such small sample numbers. Some of the current analyses were informed by observations emerging from previous studies. Furthermore, the group of PKT does not include patients transplanted below 1 year of age, making them more likely to have acquired EBV at the time of transplant. Young age at transplantation, early thymus excision, and EBV mismatch at transplantation are all interrelated and the small sample size does not allow for robust analysis.

The identified risk factors may also overlap in subjects with CHD. To address this, p-values were adjusted for multiple testing using the Benjamini–Hochberg procedure.

Some blood samples were collected very early after heart transplantation, the earliest 40 days. For the comparison with the control group of kidney-transplanted subjects, where some blood samples were collected very early, it was included in the analysis.

## Conclusions

The identified PTLD risk groups demonstrated significantly different immune patterns compared with the overall cohort. The pattern is consistent with immune dysfunction and features of immunological aging, both of which are conditions known to increase the risk of developing PTLD. We suggest close follow-up and regular evaluation of the immune system, in the previously identified risk groups and especially the group with hypoplastic left heart.

## Financial support

The study was funded by the Swedish Childhood Cancer Fund, the Swedish Heart-Lung Foundation, the Swedish Research Council, and the Agreement concerning research and education of doctors from Gothenburg University.

## Declaration of generative AI and AI-assisted technologies in the writing process

During the preparation of this work the author(s) used Copilot to improve readability and language. After using this tool/service, the author(s) reviewed and edited the content as needed and took full responsibility for the content of the publication.

## Declaration of Competing Interest

The authors declare that they have no known competing financial interests or personal relationships that could have appeared to influence the work reported in this paper.

## Footnotes

Appendix A Supplementary data associated with this article can be found in the online version at doi:10.1016/j.jhlto.2026.100673.

## References

[1] Webber S.A., Naftel D.C., Fricker F.J., Olesnevich P., Blume E.D., Addonizio L., Kirklin J.K., Canter C.E. (2006). Pediatric Heart Transplant Study. Lymphoproliferative disorders after paediatric heart transplantation: a multi-institutional study. Lancet.

[2] Hislop A.D., Taylor G.S. (2015). T-Cell Responses to EBV. Curr Top Microbiol Immunol.

[3] Gajarski R.J., Blume E.D., Urschel S., Schechtman K., Zheng J., West L.J., Altamirano L., Miyamoto S., Naftel D.C., Kirklin J.K., Zamberlan M.C., Canter C.E. (2011). Pediatric Heart Transplant Study Investigators. Infection and malignancy after pediatric heart transplantation: the role of induction therapy. J Heart Lung Transplant.

[4] Giuliano K., Canner J.K., Scully B.B., Clarke N., Fraser C.D., Ravekes W., Mettler B., Jacobs M.L., Sen D.G. (2022). Epstein-Barr Virus predicts malignancy after pediatric heart transplant, induction therapy and Tacrolimus don't. Ann Thorac Surg.

[5] Ekman-Joelsson B.-M., Wåhlander H., Synnergren M., Sager M., Mellgren K. (2017). Post-transplant lymphoproliferative disease is associated with early sternotomy and left ventricular hypoplasia during infancy: a population-based retrospective review. Cardiology Young.

[6] Tajima T., Martinez O.M., Bernstein D., Boyd S.D., Gratzinger D., Lum G., Sasaki K., Tan B., Twist C.J., Weinberg K., Armstrong B., Desai D.M., Mazariegos G.V., Chin C., Fishbein T.M., Tekin A., Venick R.S., Krams S.M., Esquivel C.O. (2024). Epstein-Barr virus-associated post-transplant lymphoproliferative disorders in pediatric transplantation: a prospective multicenter study in the United States. Pediatr Transplant.

[7] Ekman-Joelsson B.-M., Brandström P., Allén M., Andersson B., Wåhlander H., Mellgren K., Ekwall O. (2022). Immunological differences between heart- and kidney-transplanted children: a cross-sectional study. Cardiol Young.

[8] Order K.E., Rodig N.M. (2024). Pediatric kidney transplantation: cancer and cancer risk. Semin Nephrol.

[9] Fulchiero R., Amaral S. (2022). Post-transplant lymphoproliferative disease after pediatric kidney transplant. Front Pediatr.

[10] Maecker H.T., McCoy J.P., Nussenblatt R. (2012). Standardizing immunophenotyping for the human immunology project. Nat Rev Immunol.

[11] van Zelm M.C., van der Burg M., Langerak A.W., van Dongen J.J.M. (2011). PID comes full circle: applications of V(D)J recombination excision circles in research, diagnostics and newborn screening of primary immunodeficiency disorders. Front Immunol.

[12] Chen S.Y., Feng Z., Yi X. (2017). A general introduction to adjustment for multiple comparisons. J Thorac Dis.

[13] Chiu Y.F., Ponlachantra K., Sugden B. (2024). How Epstein Barr Virus causes lymphomas. Viruses.

[14] West S.C., Friedland-Little J.M., Schowengerdt K.O., Jr, Naftel D.C., Pruitt Freeze E., Smith K.S., Urschel S., Michaels M.G., Kirklin J.K., Feingold B. (2019). Characteristics, risks, and outcomes of post-transplant lymphoproliferative disease >3 years after pediatric heart transplant: a multicenter analysis. Clin Transplant.

[15] Sabourin K.R., Mugisha J., Asiki G., Nalwoga A., Labo N., Miley W., Beyer R., Rochford R., Johnston T.W., Newton R., Whitby D. (2023). Epstein-Barr virus (EBV) antibody changes over time in a general population cohort in rural Uganda, 1992-2008. Infect Agent Cancer.

[16] Opelz G., Henderson R. (1993). Incidence of non-Hodgkin lymphoma in kidney and heart transplant recipients. Lancet.

[17] Castillo J.J., Beltran B.E., Miranda R.N., Young K.H., Chavez J.C., Sotomayor E.M. (2016). EBV-positive diffuse large B-cell lymphoma of the elderly: 2016 update on diagnosis, risk-stratification, and management. Am J Hematol.

[18] Cohen J.I. (2015). Primary immunodeficiencies associated with EBV disease. Curr Top Microbiol Immunol.

[19] Gloghini A., Dolcetti R., Carbone A. (2013). Lymphomas occurring specifically in HIV-infected patients: from pathogenesis to pathology. Semin Cancer Biol.

[20] Kumar B.V., Connors T.J., Farber D.L. (2018). Human T cell development, localization, and function throughout life. Immunity.

[21] Ahmed H., Mahmud A.R., Faijanur-Rob-Siddiquee M., Shahriar A., Biswas P., Shimul E.K., Ahmed S.Z., Ema T.I., Rahman N., Khan M.A., Mizan F.R., Emran T.B. (2023). Role of T cells in cancer immunotherapy: opportunities and challenges, review article. Cancer Pathog Ther.

[22] Manlhiot C., Pollock-BarZiv S.M., Holmes C., Weitzman S., Allen U., Clarizia N.A., Ngan B.-Y., McCrindle B.W., Dipchand A.I. (2010). Post-transplant lymphoproliferative disorder in pediatric heart transplant recipients. J Heart Lung Transplant.

[23] Offor U.T., Bacon C.M., Roberts J., Powell J., Brodlie M., Wood K., Windebank K.P., Flett J., Hewitt T., Rand V., Hasan A., Parry G., Gennery A.R., Reinhardt Z., Bomken S. (2021). Transplantation for congenital heart disease is associated with an increased risk of Epstein-Barr virus-related post-transplant lymphoproliferative disorder in children. J Heart Lung Transplant.

[24] Arshad A., Azeka E., Barbar S., Marcondes R., Siqueira A., Benvenuti L., Miura N., Jatene M., Odone Filho V. (2019). Long‑term evaluation of post‑transplant lymphoproliferative disorders in paediatric heart transplantation in Sao Paulo, Brazil. Pediatr Cardiol.

[25] Karazisi C., Dellborg M., Mellgren K., Giang K.W., Skoglund K., Eriksson P., Mandalenakis Z. (2022). Risk of cancer in young and older patients with congenital heart disease and the excess risk of cancer by syndromes, organ transplantation and cardiac surgery: Swedish health registry study (1930-2017). Lancet Reg Health Eur.

[26] Karazisi C., Dellborg M., Mellgren K., Giang K.W., Skoglund K., Eriksson P., Mandalenakis Z. (2024). Outcomes after cancer diagnosis in children and adult patients with congenital heart disease in Sweden: a registry-based cohort study. BMJ Open.

[27] Perez Y.E., Moran C.A. (2022). The thymus: general concepts on embryology, anatomy, histology and immunohistochemistry. Semin Diagn Pathol.

[28] Yamagishi H. (2021). Cardiac neural crest. Cold Spring Harb Perspect Biol.

[29] Bremer S.J., Boxnick A., Glau L., Biermann D., Joosse S.A., Thiele F., Billeb E., May J., Kolster M., Hackbusch R., Fortmann M.I., Kozlik-Feldmann R., Hübler M., Tolosa E., Sachweh J.S., Gieras A. (2024). Thymic atrophy and immune dysregulation in infants with complex congenital heart disease. J Clin Immunol.

[30] Kumar B.V., Connors T.J., Farber D.L. (2018). Human T cell development, localization, and function throughout life. Immunity.

[31] Halnon N.J., Jamieson B., Plunkett M., Kitchen C.M., Pham T., Krogstad P. (2005). Thymic function and impaired maintenance of peripheral T cell populations in children with congenital heart disease and surgical thymectomy. Pediatr Res.

[32] Ogle B.M., West L.J., Driscoll D.J., Strome S.E., Razonable R.R., Paya C.V., Cascalho M., Platt J.L. (2006). Effacing of the T cell compartment by cardiac transplantation in infancy. J Immunol.

[33] Mengrelis K., Kucera F., Shahid N., Watt E., Ross S., Lau C.I., Adams S., Gilmour K., Pils D., Crompton T., Burch M., Davies E.G. (2021). T cell phenotype in paediatric heart transplant recipients. Pediatr Transplant.

[34] Silva S.L., Albuquerque A., Amaral A.J., Li Q.Z., Mota C., Cheynier R., Victorino R.M.M., Pereira-Santos M.C., Sousa A.E. (2017). Autoimmunity and allergy control in adults submitted to complete thymectomy early in infancy. PLoS One.

[35] Gudmundsdottir J., Söderling J., Berggren H., Óskarsdóttir S., Neovius M., Stephansson O., Ekwall O. (2018). Long-term clinical effects of early thymectomy: associations with autoimmune diseases, cancer, infections, and atopic diseases. J Allergy Clin Immunol.

[36] Avdimiretz N., Seitz S., Kim T., Murdoch F., Urschel S. (2018). Allergies and autoimmune disorders in children after heart transplantation. Clin Transplant.

[37] Kooshesh K.A., Foy B.H., Sykes D.B., Gustafsson K., Scadden D.T. (2023). Health consequences of thymus removal in adults. N Engl J Med.

[38] Deya-Martinez A., Flinn A.M., Gennery A.R. (2020). Neonatal thymectomy in children-accelerating the immunologic clock?. J Allergy Clin Immunol.

[39] Terekhova M., Bohacova P., Artyomov M.N. (2025). Human immune aging. Immunity.

