# Supplementary material for: Risk factors for posttransplant lymphoproliferative disorder (PTLD) in pediatric heart recipients were associated with impairment of the immune system – a multicenter study from Sweden
Source: JHLT Open. 2026 Aug 27;14:100673. doi: 10.1016/j.jhlto.2026.100673 (PMC13586574; doi:10.1016/j.jhlto.2026.100673)
Supplement: Supplementary file 1 [file mmc1.docx]

Supplementary material for the article:

Methods

Flow cytometry was used to determine the concentrations of subpopulations of lymphocytes and myeloid cells in the blood (Table 1). To determine the concentrations of NK, B, CD4+ and CD8+ T cells, 50 µL of whole blood were stained with the BD Multitest 6-color TBNK reagents in Trucount tubes (BD Biosciences, Mountain View, CA, USA). Trucount tubes were also used to determine the concentrations of monocytes using antibodies against CD45 (fluorescein isothiocyanate, FITC-conjugated) and CD14 (phycoerythrin, PE-conjugated). For other panels, 1 mL of blood collected in an EDTA-coated vacutainer tube was washed twice with 2 mL of FACSFlow (BD Biosciences), followed by centrifugation for 5 minutes at 300 × *g* to remove serum antibodies. After resuspension in 1 mL of FACSflow, 100 µL of the cells were mixed with pre-mixed stock solutions of antibodies using the concentrations recommended by the manufacturer. The respective tubes were stained with conjugated antibodies against: (i) CD31 (FITC), CCR7 (PE), CD4 (peridinin-chlorophyll-protein complex Cy5.5, PerCP-Cy5.5), CD45RA (PE-Cy7), CD38 (allophycocyanin, APC), CD8 (APC-Cy7), CD3 (V450), and HLA-DR (V500-C); (ii) CCR7 (FITC), CD25 (PE), CD4 (PerCP-Cy5.5), CCR4 (PE-Cy7), CD127 (APC), CD45R0 (APC-H7), CD3 (V450), and HLA-DR (V500-C); (iii) CXCR5 (BB515), CXCR3 (PE), CD4 (PerCP-Cy5.5), CCR6 (PE-Cy7), CCR4 (Alexa Fluor 647), CD45R0 (APC-H7), CD3 (V450), and HLA-DR (V500-C); (iv) IgM (BB515), CD24 (PE), CD19 (PerCP-Cy5.5), CD27 (PE-Cy7), CD38 (APC), CD20 (APC-H7), CD21 (V450), and IgD (V500-C); and (v) CD56 (PE), CD123 (PerCP-Cy5.5), CD11c (PE-Cy7), CD16 (APC), CD3+CD19+CD20 (APC-H7), CD14 (V450), and HLA-DR (V500-C) [all antibodies were from BD Biosciences, except for CD127-APC, which was from Thermo Fisher Scientific (Waltham, MA, USA) and CD56-PE (Beckman Coulter, Atlanta, GA, USA)]. Cell subtypes were identified using the gating strategy described by Maecker et al. ^10^ After incubation at room temperature for 15 minutes, the cells were washed, lysed and fixed using FACS Lyse Wash Assistant (BD Biosciences). Cell analysis was performed in the FACSCanto II flow cytometer (BD Biosciences) using the FACSDiva software. A lymphocyte gate was defined based on the location in a CD45 versus side-scatter. During the setting up of the assay, fluorescence-minus-one (FMO) controls were run to ensure that the quadrant delineated positive fluorescent staining. To determine the subpopulation concentrations, the results from the Trucount tubes were multiplied by the cell counts from the other tubes. The results for each subpopulation are expressed as the percentage of lymphocytes and as the number of cells × 10^9^/L.

To determine the numbers of T-receptor excision circles (TREC), genomic DNA was extracted using the QIAamp DNA Blood Mini Kit (Qiagen, Hilden, Germany) from 200 µL of whole blood that was collected in EDTA tubes, as described by the manufacturer. The purified DNA concentrations were assessed fluorometrically (Quantus®; Promega, Madison, WI, USA) and adjusted to 30 ng/μL with DNase/RNase-free H_2_O (Gibco, no. 10977 Thermo Fisher Scientific). Real-Time PCR was performed in triplicate in a Roche Light Cycler 480 II instrument to detect the numbers of copies of *TREC* and the control gene *GAPDH* (for glyceraldehyde-3-phosphate dehydrogenase). The following primers (forward and reverse, respectively) were used (Thermo Fisher Scientific): for *TREC*, 5′-CAT CCC TTT CAA CCA TGC TGA CAC CTC T-3′ and 5′-CGT GAG AAC GGT GAA TGA AGA GCA GAC A-3′; and for *GAPDH*, 5′-CAG CCC CTT CAT ACC CTC A-3′ and 5′-GGA CCA TAT TGA GGG ACA CA 3′. The PCR-mix contained 10 μL LightCycler® 480 SYBR Green I Master (Roche), 3 μL sample or controls (dH_2_O as negative control and 30 ng/µL of cord blood as positive control), 2 × 1.4 μL primer solution (forward and reverse primers, 0.5 μM each), and 4.2 μL dH_2_O. The results for each sample were calculated by extrapolating the absolute concentration from standard curves constructed with known dilutions (108- to 101-fold) of the pCR2.1-human *TREC* or pCR2.1-*GAPDH* gene plasmids (Eurofins MWG Operon; Eurofins Genomics GmbH, Ebersberg, Germany) with fixed concentrations of the *TREC* plasmid and *GAPDH* plasmid as curve adjustors. The formula: (*TREC* copies)/(½×*GAPDH* copies) was used to determine the concentrations of *TREC* transcripts in white blood cells.

Table S1. Characteristics of the different immune cell types.

| **Cell type** | **Cell surface marker(s)** |
| --- | --- |
|  |  |
| **T lymphocytes** | **CD3+** |
| **T helper lymphocytes** | **CD3^+^CD4^+^** |
| Naïve T helper lymphocytes | CD3^+^CD4^+^CCR7^+^CD45RA^+^ |
| Recent thymic emigrants (RTEs) | CD3^+^CD4^+^CCR7^+^CD45RA^+^CD31^+^ |
| Central memory T helper lymphocytes | CD3^+^CD4^+^CCR7^+^CD45RA^-^ |
| Effector T helper lymphocytes | CD3^+^CD4^+^CCR7^-^CD45RA^+^ |
| Effector memory T helper lymphocytes | CD3^+^CD4^+^CCR7^-^CD45RA^-^ |
| Effector memory T helper lymphocytes (EMRA) | CD3^+^CD4^+^CCR7^-^CD45RA^low^ |
| Activated central memory T helper lymphocytes | CD3^+^CD4^+^CCR7^+^CD45RA^-^CD38^+^HLA DR^+^ |
| Activated effector memory T helper lymphocytes | CD3^+^CD4^+^CCR7^-^CD45RA^-^CD38^+^HLA DR^+^ |
| Th1 | CD3^+^CD4^+^CCR7^+^CD45RA^-^CXCR3+CCR6- |
| Th2 | CD3^+^ CD4^+^CXCR3^+^CCR6^-^ |
| Th17 | CD3^+^CD4^+^CXCR3^-^CCR6^+^ |
| Follicular T helper cells | CD3^+^CD4^+^CD45RO^+^CXCR3^-^CCR6^-^CCR4^-^CXCR5^+^ |
| Naïve T regulatory lymphocytes | CD3^+^CD4^+^CCR4^+^CD25^hi^CD127^low^CD45RO^-^ |
| Memory T regulatory lymphocytes | CD3^+^CD4^+^CCR4^+^CD25^hi^CD127^low^CD45RO^+^ |
|  |  |
| **T cytotoxic lymphocytes** | **CD3^+^CD8^+^** |
| Naïve T cytotoxic lymphocytes | CD3^+^CD8^+^CCR7^+^CD45RA^+^ |
| Central memory T cytotoxic lymphocytes | CD3^+^CD8^+^CCR7^+^CD45RA^-^ |
| Effector memory T cytotoxic lymphocytes | CD3^+^CD8^+^CCR7^-^CD45RA^-^ |
| Activated effector memory T cytotoxic lymphocytes | CD3^+^CD8^+^CCR7^-^CD45RA^-^CD38^+^HLA DR^+^ |
| Effector memory T cytotoxic lymphocytes (EMRA) | CD3^+^CD8^+^CCR7^-^CD45RA^low^CD38^+^HLA DR^+^ |
|  |  |
| **B lymphocytes** | **CD3^-^CD19^+^** |
| Naïve B lymphocytes | CD3^-^CD19^+^CD20^+^CD27^-^CD24^low^CD38^low^IgD^+^IgM^+^ |
| Transitional B lymphocytes | CD3^-^CD19^+^CD20^+^CD27^-^CD24^hi^CD38^hi^IgD^+^IgM^+^ |
| Marginal zone-like B lymphocytes | CD3^-^CD19^+^CD20^+^CD27^+^IgD^+^IgM^+^ |
| IgM+ memory B lymphocytes | CD3^-^CD19^+^CD20^+^CD27^+^IgD^-^IgM^+^ |
| Class-switched memory B lymphocytes | CD3^-^CD19^+^CD20^+^CD27^+^IgD^-^IgM^-^ |
| Plasmablasts | CD3^-^CD19^+^CD20^-^CD38^+^CD27^hi^ |
|  |  |
| **NK cells** | **CD3-CD56^+^** |
|  |  |
| **Monocytes** | **CD14^+^** |
| **Dendritic cells** | **CD3^-^CD19^-^CD20^-^CD14^-^** |

Table S2. Immune suppression for heart transplanted versus kidney transplanted children

|  |  | Heart-transplanted (N=76) | | Kidney-transplanted (N=34) | |
| --- | --- | --- | --- | --- | --- |
|  | Drug | Fraction of patients  Body weight: dose regimen | Target range, ng/mL | Fraction of patients.  Body weight: dose regimen | Target range, ng/mL |
| Induction  treatment | ATG-F 2007–2015 | 24/76 patients |  | 0/36 patients |  |
|  | Thymoglobin | 27/76 |  |  |  |
|  | Basiliximab | 25/76 patients  <35 kg: 10 mg Day 0 and Day 4  >35 kg: 20 mg Day 0 and Day 4 |  | 25/34 patients  <35 kg: 10 mg Day 0 and Day 4  >35 kg: 20 mg Day 0 and Day 4 |  |
|  | Steroids | 10 mg/kg Day 0 – weaning over 2 days to 0.2 mg/kg daily |  | 34/34  600 mg/m^2^ Day 0,  weaning over 28 days |  |
| Initial treatment  (0–6 months) | Tacrolimus | 76/76 patients  0.1 mg/kg twice daily | 10–12 | 34/34  0.1 mg/kg twice daily | 5–8 |
|  | Mycophenolic acid | 76/76  300 mg/m^2^ twice daily |  | 34/34  300 mg/m^2^ twice daily |  |
|  | Steroids | 76/76 patients  0.2 mg/kg once  daily |  | 34/34  From day 29: 10 mg/m^2^ every other day |  |
| Treatment during blood sample | Tacrolimus  Mycophenolic acid  Everolimus  Everolimus+Ciklosporine  Steroids | 68/76 patients  47/76 patients  16/76 patients  5/76  7/76 | 4-8 | 33/34 patients  25/34 patients  1/34 patients  31/34 patients | 4-8 |
